# [Expression of Concern] Fatty acids inhibit anticancer effects of 5-fluorouracil in mouse cancer cell lines

**DOI:** 10.3892/ol.2026.15756

**Published:** 2026-07-09

**Authors:** Eriko Tanabe, Misaho Kitayoshi, Kiyomu Fujii, Hitoshi Ohmori, Yi Luo, Yui Kadochi, Shiori Mori, Rina Fujiwara, Yukiko Nishiguchi, Takamitsu Sasaki, Hiroki Kuniyasu

Oncol Lett 14: 681–686, 2017; DOI: 10.3892/ol.2017.6190

Following the publication of the above paper, it has been drawn to the Editor's attention by a concerned reader that, regarding the experiments showing the expression of CD133 (Fig. 2A) and nucleostemin (NC) (Fig. 2C) on p. 684, the ACTB data for the CT26 and CMT93 cell lines in both figure parts appeared to be matching, suggesting that this figure may have been assembled incorrectly.

The authors have been contacted by the Editorial Office to offer an explanation for this apparent anomaly in the presentation of the data in this paper, and we are awaiting their response. Owing to the fact that the Editorial Office has been made aware of this potential issue surrounding the scientific integrity of this paper, we are issuing an Expression of Concern to notify readers of this potential problem while the Editorial Office continues to investigate this matter further.

